# Locally advanced breast cancer patients should be cautious about the immediate breast reconstruction after mastectomy: a pooling analysis of safety and efficacy

**DOI:** 10.1186/s12957-024-03444-z

**Published:** 2024-06-25

**Authors:** Cheng-Yu Zeng, Yan-Yu Qiu, Jia-Yi Li, Jian-Hao Huang, Xue-Song Bai, Xian-Lin Han, Xiao-Dong He

**Affiliations:** 1 Eight-Year Program of Clinical Medicine, Chinese Academy of Medical Sciences, Peking Union Medical College Hospital, Peking Union Medical College, Beijing, China; 2 Department of General Surgery, State Key Laboratory of Complex Severe and Rare Diseases, Peking Union Medical College Hospital, China Academy of Medical Science & Peking Union Medical College, 1 Shuaifuyuan, Dongcheng District,, Beijing, China

**Keywords:** Locally advanced breast cancer, Immediate breast reconstruction, Overall survival, Surgical complication, Meta-analysis

## Abstract

**Background:**

The purpose of this study was to compare safety and efficacy outcomes between immediate breast reconstruction (IBR) and mastectomy alone in locally advanced breast cancer patients.

**Methods:**

We conducted a comprehensive literature search of PUBMED, EMBASE, and Cochrane databases. The primary outcomes evaluated were overall survival, disease-free survival, and local recurrence. The secondary outcome was the incidence of surgical complications. All data were analyzed using Review Manager 5.3.

**Results:**

Sixteen studies, involving 15,364 participants were included in this meta-analysis. Pooled data demonstrated that patients underwent IBR were more likely to experience surgical complications than those underwent mastectomy alone (HR: 3.96, 95%CI [1.07,14.67], *p* = 0.04). No significant difference was found in overall survival (HR: 0.94, 95%CI [0.73,1.20], *p* = 0.62), disease-free survival (HR: 1.03, 95%CI [0.83,1.27], *p* = 0.81), or breast cancer specific survival (HR: 0.93, 95%CI [0.71,1.21], *p* = 0.57) between IBR group and Non-IBR group.

**Conclusions:**

Our study demonstrates that IBR after mastectomy does not affect the overall survival and disease-free survival of locally advanced breast cancer patients. However, IBR brings with it a nonnegligible higher risk of complications and needs to be fully evaluated and carefully decided.

**Supplementary Information:**

The online version contains supplementary material available at 10.1186/s12957-024-03444-z.

## Introduction

Breast cancer is the most common cancer and the second leading cause of cancer death, and the incidence rates have risen in most recent years by 0.5% annually [1]. Mastectomy is a common and effective treatment for breast cancer patients, while mastectomy not only leads to various physical discomfort, including loss of sensation, impaired body image, and sexual function but also impairs mental health [2, 3]. Research has shown that immediate breast reconstruction (IBR) after mastectomy can restore body image and preserve femininity [4], breast cancer survivors underwent IBR generally report better quality of life in psychosocial, sexual, and physical well-being domains [5, 6], especially in younger women. In addition, immediate autologous reconstruction could preserve the natural skin envelope and relatively reducing scar formation, reduce the adhesion of the skin paddle to the chest wall with improved cosmesis [7, 8]. IBR after breast cancer mastectomy is on the rise because of its positive impact on patient’s quality of life over the past 10 years [9–11], while it is generally limited to patients with low-risk diseases, with significantly lower use for higher tumor stages (*p* < 0.0001), despite these potential benefits [12].

Locally advanced breast cancer (LABC) refers to a large category of breast cancer with extensive lesion invasion or regional lymph node metastasis, without distant metastasis, including clinical stage IIB to IIIC. Modest improvement in survival or QoL (Quality of Life) was achieved in recent years underscoring the unmet need in LABC patients [13]. The surgical complexity and high-risk oncological characteristics make IBR a huge challenge for LABC patients. In addition, comprehensive treatment including neoadjuvant chemoradiotherapy and post-mastectomy radiotherapy is becoming the new normal for LABC patients [14–16], therefore, the technical feasibility of IBR has to be taken into consideration.

To date, it is gradually accepted that LABC is no longer contraindicated for breast reconstruction. There are different views on whether IBR should be performed. Some retrospective studies report IBR has a survival advantage over mastectomy for possible antitumorigenic effect of implants [16], and neither increases the risk of local recurrence (LR) nor delays adjuvant therapy [17]. But there’s also report of IBR associating with a worse outcome of local control [18]. Additionally, a risk assessment nomogram has been developed to broaden the application of IBR in LABC patients, allowing surgeons to recommend IBR through preoperative evaluation. Identified risk factors include older age, unmarried status, and more advanced stage [19]. However, this study focused solely on oncological outcomes, and many surgeons are also concerned about the potential increased risk of complications associated with IBR in LABC patients. Therefore, we performed a meta-analysis comparing overall survival, disease-free survival, local recurrence, and surgical complications in LABC patients who underwent IBR after mastectomy and mastectomy alone.

## Methods and materials

This meta-analysis was conducted according to the Preferred Reporting Items for Systematic Reviews and Meta-analyses (PRISMA) standards [20], and the protocol was registered in the PROSPERO database (CRD42024501216).

### Data sources

Two independent reviewers conducted searched of the PubMed, Embase, and Cochrane Library databases from inception to December 2023 for relevant English articles. Reviewers manually evaluated full texts of studies based on the prespecified inclusion criteria and exclusion criteria, and all references were managed in an Endnote database (Version 19.0). Any discrepancies were resolved by discussion and got consensus with a third reviewer.

### Study selection

Studies were eligible for the meta-analysis if they fulfilled the inclusion criteria: (1) retrospective or prospective cohort studies; (2) comparison between IBR (IBR-group) with mastectomy alone (Non-IBR group) in LABC female patients; (3) report at least one of the outcome indicators. Studies were excluded if they were: (1) conference abstract, letters, editorials opinions, meta-analysis, or reviews. (2) single-arm cohort studies. Two studies [21, 22] both used SEER database but included different population and were both included in our analysis.

### Risk of bias (quality) assessment

Reviewers assessed the quality of included studies using the Newcastle-Ottawa Scale (NOS). More than six stars were considered to be of high quality. Two reviewers independently assessed the quality of all included studies by selection, comparability and exposure. Discrepancies were re-examined by a third reviewer, and a consensus was reached by discussion.

### Data extraction

Reviewers (CY.Z., JY.L.) independently extracted data from included studies into tables including study region, publication year, Enrollment time span, study design, participant characteristics, surgical characteristics, oncology outcomes, and surgical complications. Additional reviewer (YY.Q.) reviewed data for accuracy. Oncology outcomes includes overall survival (OS), disease-free survival (DFS), breast cancer specific survival (BCSS), and local recurrence (LR). Local recurrence was defined as any recurrence in the ipsilateral mastectomy site. Surgical complications were classified as major complications and minor complications, of which major complications were defined as complications requiring reoperation or other nonconservative subsequent treatment. Reconstruction methods are categorized into autologous and alloplastic reconstruction. Autologous reconstruction utilizes the patient’s own tissue, whereas alloplastic reconstruction predominantly employs various types of implants. All data were extracted directly from publications, and contacting authors for additional information was not necessary. and contacting authors for additional information was not necessary.

### Data synthesis

A pooled data synthesis was performed by the Review Manager (RevMan Version 5.4.). For observation-event outcomes (including positive margins, LR, and surgical complications), odds ratios (OR) and 95% CIs of events for IBR to Non-IBR were retrieved or calculated. For time-to-event outcomes (including OS, DFS, BCSS), hazard ratios (HR) were pooled according to the inverse of variance method. If the survival curves of OS, DFS, and BCSS were reported in the literature, the survival data were extracted using Engauge Digitizer version 4.1 software and then calculated using the Excel attachment calculations spreadsheet provided by Tierney et al [23].

Statistically significant heterogeneity was defined as a P value less than 0.1 or an I² statistic greater than 50%. In the absence of heterogeneity, pooled estimates of ORs or HRs with their 95% CIs were calculated using the Mantel-Haenszel method. In the presence of heterogeneity, the Der Simonian and Laird random effects method were used to pool primary study estimates. All significance testing was two-sided, and results were considered statistically significant if *P* < 0.05. Funnel plots were presented as an assessment tool for publication bias.

## Results

**Fig. 1:**
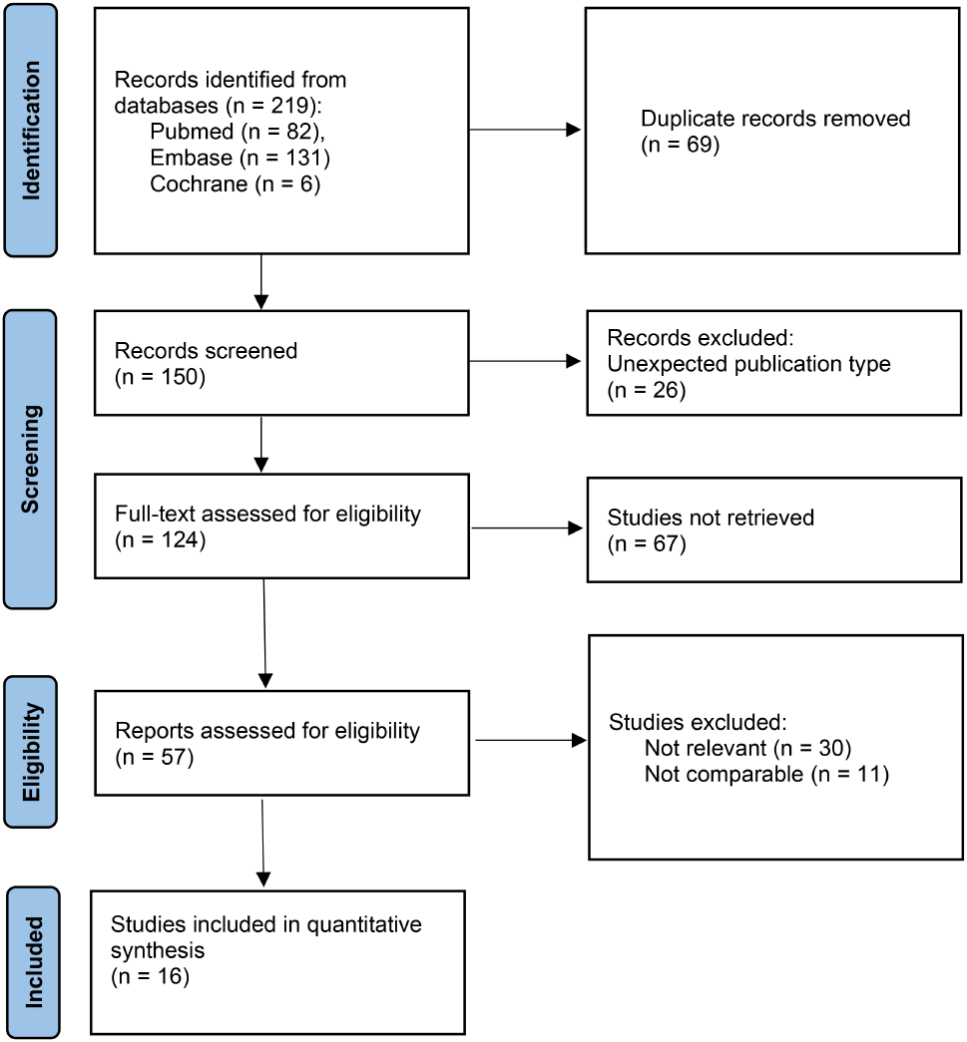
Flowchart of study selection process

A total of 16 retrospective studies involving 15,364 participants (4135 patients underwent IBR, 11,229 patients underwent mastectomy alone) met inclusion criteria and provided survival or surgical complication outcomes for the meta-analysis (Fig. 1) [22, 24–38]. Studies were mostly conducted in North America, with additional studies in Asia and Europe (Table 1). Ten articles were published in the last five years contributing to good applicability.

**Table 1:** Basic characteristics and the quality evaluation of the studies included

Author	Year	Year of entry	Country	Study design	Sample size			Stage (IIB/III*)		NOS**
					Total	IBR	Non-IBR	IBR	Non-IBR	
Newman LA	1999	1990-1993	USA	Retrospective	122	50	72	23/27	12/60	7
Rey P	2005	1999-2002	Italy	Retrospective	105	90	15	-	-	7
Lim W	2010	1996-2005	Korea	Retrospective	897	87	810	8/79	54/756	8
Prabhu R	2012	1999-2010	USA	Retrospective	100	40	60	4/36	3/57	7
Hsieh TY	2014	2002-2009	China	Retrospective	192	52	140	0/52	0/140	8
Wu SG	2018	2003-2010	USA	Retrospective(PSM)	3464	1732	1732	-	-	8
Da Costa Vieira RA	2019	2005-2011	Brazil	Retrospective	144	48	96	7/41	7/89	8
Wang M	2019	1998-2015	USA	Retrospective(PSM)	1473	491	982	0/491	0/982	8
Yoon WS	2019	2007-2014	Korea	Retrospective	188	61	127	40/221	71/56	7
Stein MJ	2020	2014-2018	Canada	Retrospective	60	37	23	-	-	8
Dudley CM	2021	2006-2007	USA	Retrospective	5318	692	4626	483/209	2922/1704	8
Taqi K	2021	2012-2017	Canada	Retrospective	267	112	155	52/60	82/73	9
Di Leone A	2022	2016-2021	Italy	Retrospective	297	210	87	-	-	8
Tomita S	2022	2005-2019	Japan	Retrospective	500	120	380	-	-	7
Wu ZY	2022	2010-2016	Korea	Retrospective(PSM)	418	209	209	109/100	100/109	8
Sang Y	2023	2010-2019	China	Retrospective	1819	104	1715	63/41	889/826	9

PSM: Propensity Score Matching. IBR: immediate breast reconstruction. *Tumor stage III includes IIIa, IIIb, and IIIc. ** Quality assessment of the observation studies was assessed using the NOS. The quality of the evidence is classified as three levels: high (more than seven stars), moderate (four to six stars), poor (less than four stars)

Pooled information was shown in Table 2. The median age of the IBR group was significantly younger than Non-IBR group (Mean difference = -7.78, 95%CI [-9.95, -5.61], *p* < 0.0001). Patients in Non-IBR group were likely to have more advanced clinical tumor stages than those in IBR group (*p* < 0.001). Tripe negative breast cancer patients were more common in Non-IBR group (OR = 0.77, 95%CI [0.64,0.92], *p* = 0.005). There were no significant differences between the two groups in histology grade (OR = 0.99, 95%CI [0.77,1.28], *p* = 0.94) (Table 2, Supplementary Figure S1).

**Table 2:** Characteristics of studies included in the meta-analysis

Characteristics	Studies	IBR/Non-IBR	Mean difference/Odds ratio (95% CI)	*p*	Heterogeneity
Age	9	770/1782	-7.78[-9.95, -5.61]	*p*<0.00001	I²=86%, *P* <0.00001
Tumor stage	9		1.34[1.18,1.53]	*p*<0.001	I²=43%, *P* = 0.08
IIB		789/4140			
III		614/3730			
Molecular types	9		0.77[0.64,0.92]	*p*=0.005	I²=0%, *P* = 0.86
TN		197/1109			
Non-TN		1316/6001			
Histology grade	11		0.99[0.77,1.28]	*p*=0.94	I²=83%, *P* <0.00001
I+II		1793/5018			
III		1706/3859			
ALND	3	(270/425)/(1836/2079)	0.52[0.39,0.69]	*p*<0.0001	I²=22%, *P* = 0.28
Positive margins	4	(18/479)/(17/645)	1.79[0.87,3.69]	*p*=0.11	I²=0%, *P* = 0.89
Complications	8	(155/558)/(296/928)	4.77[1.12,20.27]	*p*=0.03	I²=89%, *P* <0.00001
Major complications	4	(43/255)/(14/571)	5.14[1.69,15.61]	*p*=0.004	I²=57%, *P* =0.07
Minor complications	5	(101/295)/(282/631)	3.22[0.47,20.00]	*p*=0.23	I²=94%, *P* <0.00001
Survival information					
OS	9		0.95[0.86,1.06]	*p* =0.36	I²=42%, *P* = 0.09
DFS	6		1.01[0.82,1.26]	*p* = 0.91	I²=0%, *P* = 0.74
BCSS	4		0.93[0.71,1.21]	*p* = 0.57	I²=63%, *P* = 0.04
LR	12		1.12[0.79,1.59]	*p* = 0.53	I²=28%, *P* = 0.17

ALND: axillary lymph node dissection. OS: overall survival. DFS: disease free survival. BCSS: breast cancer specific survival. LR: local recurrence

### Oncological outcomes

**Fig. 2:**
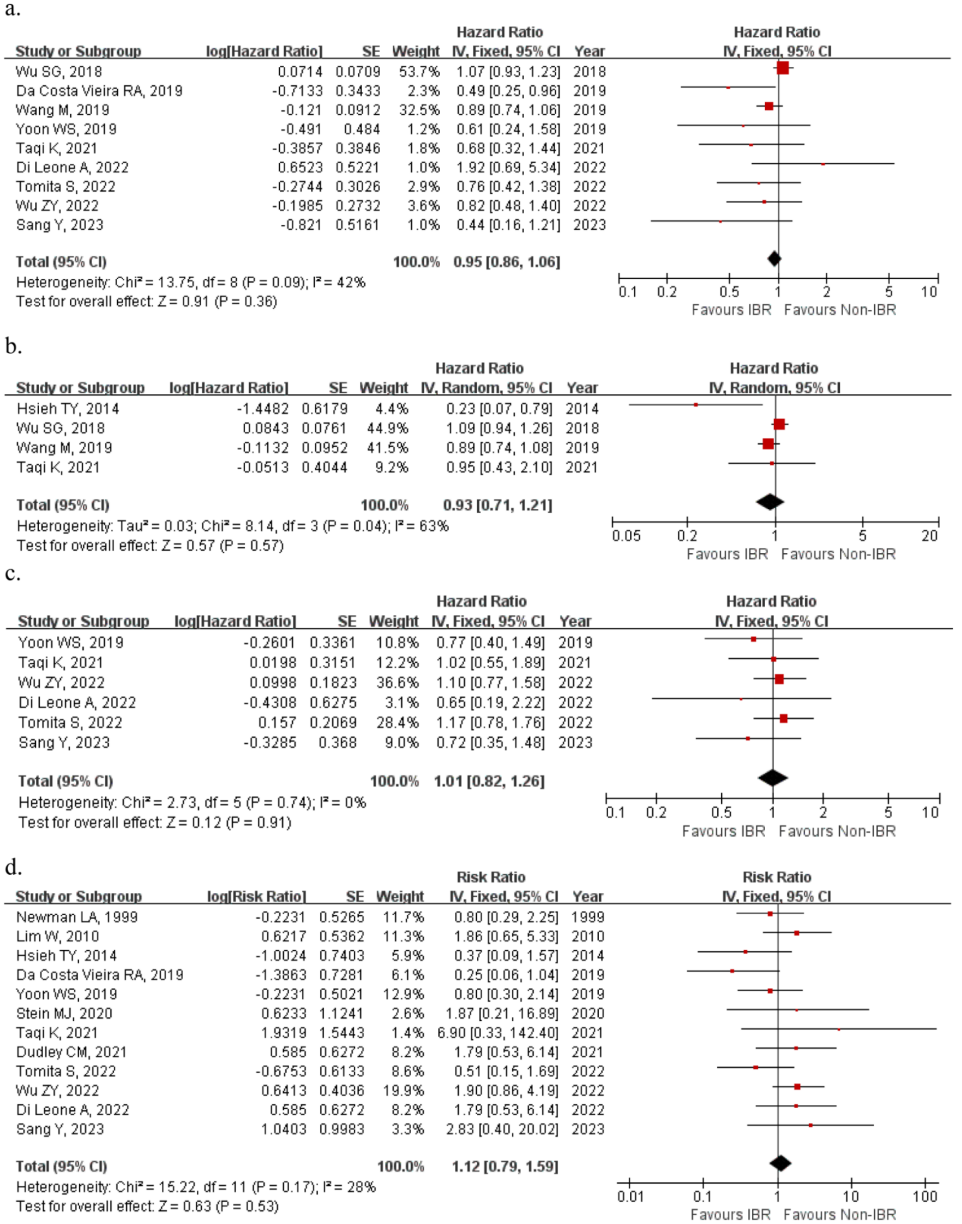
Forest plot of survival information included in the study. (**a**) OS; (**b**) BCSS; (**c**) DFS; (**d**) LR

Overall survival was reported in 9 studies, pooled HR data demonstrated that patients underwent IBR were associated with better overall survival than those in Non-IBR group, but the difference was not significant (HR: 0.95, 95%CI [0.86,1.06], *p* = 0.36). Similarly, there’s no significant difference in DFS (HR: 1.01, 95%CI [0.82,1.26], *p* = 0.91) and BCSS (HR: 0.93, 95%CI [0.71,1.21], *p* = 0.57) between IBR group and Non-IBR group. None of the included studies showed a statistically significant difference in DFS between study groups. The pooled OR for LR was 1.12 (95% CI: [0.79, 1.59], *p* = 0.53) as shown in Fig. 2d. there were no significant differences in the frequency of LR in patients treated with IBR compared with those who underwent mastectomy alone. Taqi et al. reported a relatively higher local recurrence (1.9% vs. 0%; RR: 6.90, 95% CI: [0.33,142.40]) with a relatively more advanced tumor stage in IBR group.

### Surgical process

**Fig. 3:**
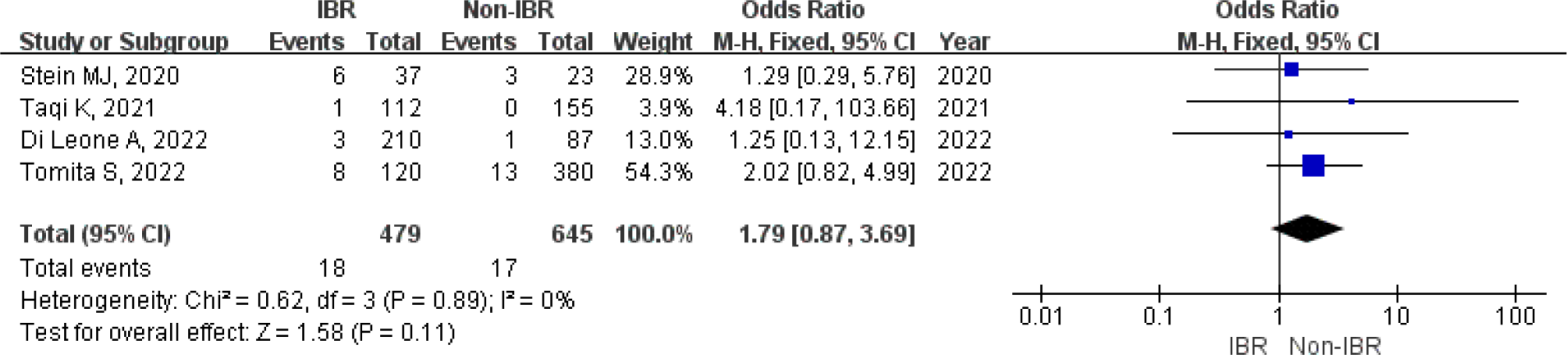
Forest plot of positive margin rate

As expected, axillary lymph node dissection (ALND) was less common in patients underwent IBR because of the earlier N stage. Interestingly, the concern of a higher positive margin rate due to additional surgical process in IBR group was not found (HR: 1.79, 95%CI [0.87,3.69], *p* = 0.11) (Fig. 3).

### Surgical complications

**Fig. 4:**
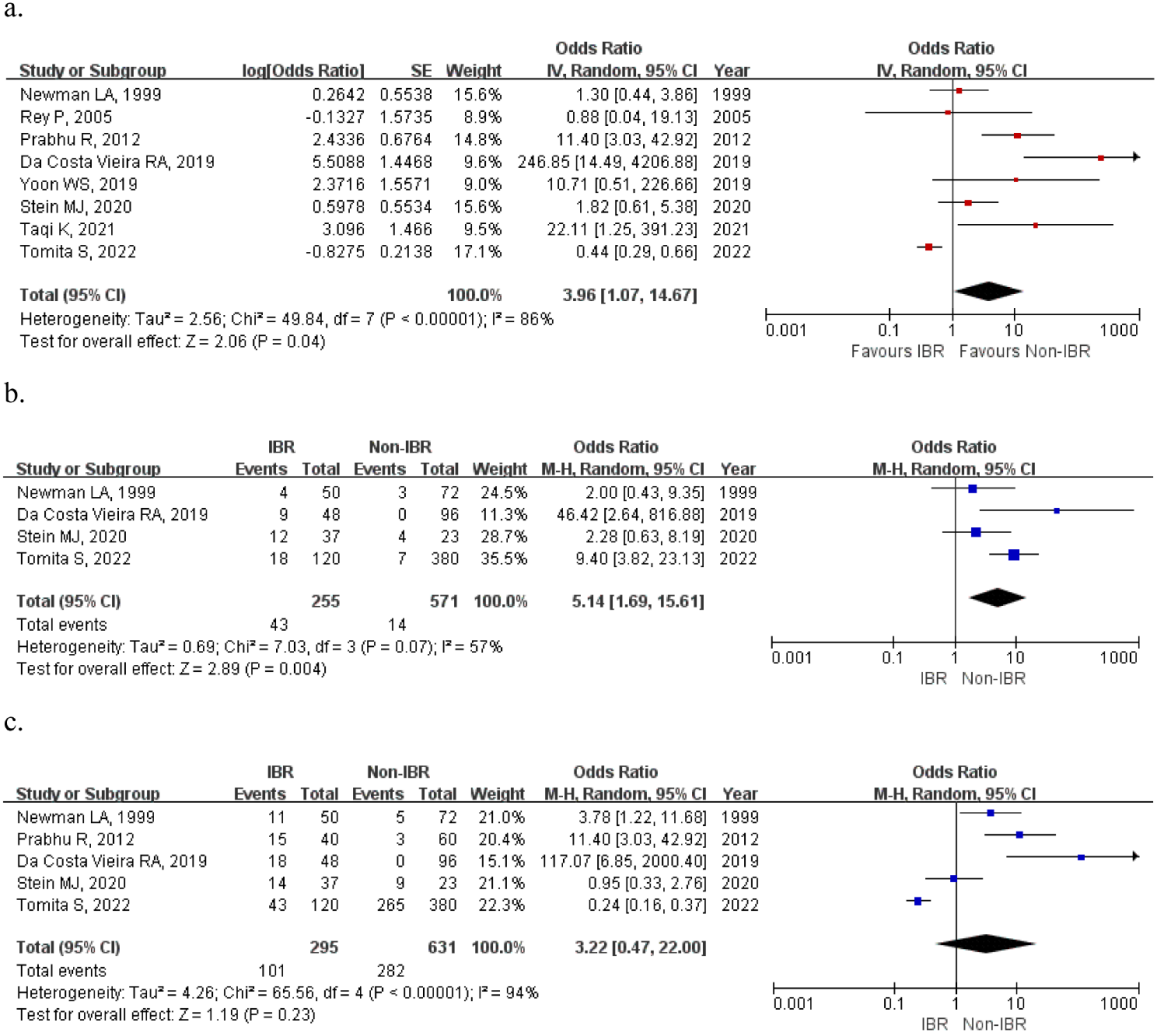
Forest plot of postoperative complication included in the study. (**a**) Total complication; (**b**) Major complication; (**c**) Minor complication

It is noteworthy that patients in IBR group had a significantly higher surgical complication risk than patients underwent mastectomy alone (HR: 3.96, 95%CI [1.07,14.67], *p* = 0.04), mainly due to a significantly higher major complication rate (HR: 5.14, 95%CI [1.69,15.61], *p* = 0.004). In terms of minor complications, the rates of the two groups were comparable (HR: 3.22, 95%CI [0.47,22.00], = 0.23) (Fig. 4).

### Survival outcomes in PSM studies

**Fig. 5:**
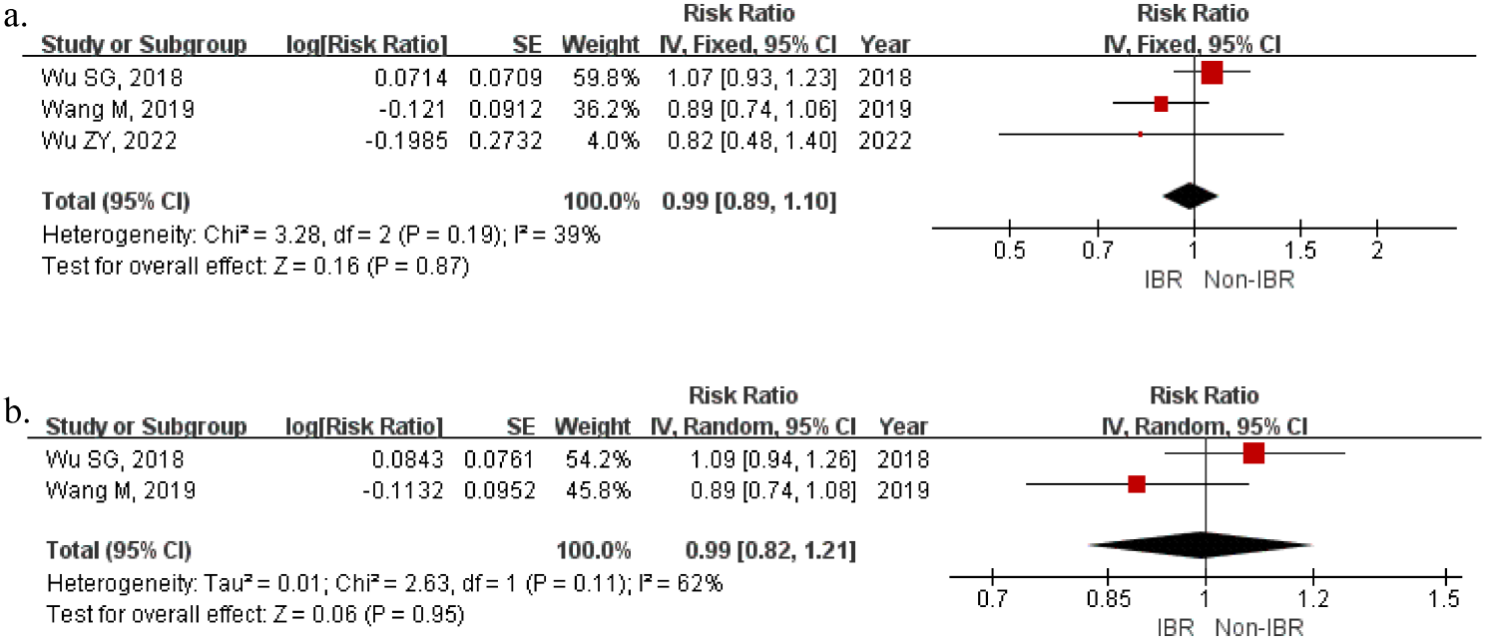
Survival of studies using PSM. (**a**) OS; (**b**) BCSS

We also conducted an analysis comprised of 3 matched cohort studies separately, where patients who had undergone IBR were matched to patients of similar age and tumor stage undergoing mastectomy without IBR (Supplementary, Table S1). There were a total of 2432 pairs. There were no significant differences between the two groups in histology grade (OR = 0.99, 95%CI [0.89,1.11] *p* = 0.89). After matching, the OS and BCSS of the IBR group and the Non-IBR group were almost identical (OS: HR 0.99, 95%CI [0.89,1.10], *p* = 0.87; BCSS: HR 0.99, 95%CI [00.82,1.21], *p* = 0.95) (Fig. 5).

### Publication bias

An assessment of publication bias indicated no small study effects for OS, BCSS, LR, and surgical complications.

## Discussion

Prior studies reported a satisfactory oncological safety of IBR in all-stage populations [38], while our meta-analysis was the first to concentrate on LABC patients and to demonstrate the oncological outcomes based on a relatively large sample size, to reveal the surgical complication risks.

Compared with mastectomy alone, IBR was significantly associated with higher total surgical complication and major complication risks in this meta-analysis. Of 8 studies reporting complication rates, Tomita et al. was the only one to describe a significantly lower complication rate in the IBR group [35]. In effect, in this single-center retrospective study, only seroma was reported more frequently in patients without breast reconstruction, other complications still dominate in IBR group, which may be the implications of reporting bias.

Major complication usually brings more hospital stays, which will increase the duration of adjuvant therapy and affect patient therapeutic experiences [35]. Common complications of IBR include surgical site infection, flap necrosis, and seroma. The possible reason is that the operation technique of IBR is more complicated with longer operation times. Surgeons are also concerned that post-surgical complications could delay the timely administration of therapies. However, two articles that examined the interval until adjuvant therapy administration reported that the differences in timing were statistically nonsignificant, even when complications occurred more frequently following reconstruction [24, 35].

IBR could be broadly divided into autologous reconstruction and alloplastic reconstruction, latissimus dorsi flap and transverse rectus abdominus myo-cutaneous are commonly used in autologous reconstruction, and alloplastic reconstruction mainly contains various implants.

For alloplastic reconstruction, implantation materials pose a greater risk of infection, and expander or recurrent infections leading to expander or implant loss can impede further therapy.

This is particularly problematic for delayed reconstruction, where infections cause inflammation and scarring, delaying subsequent surgeries. Furthermore, inflammation and capsule formation caused by expanders in delayed-reconstruction make preparing the flap recipient site technically more challenging [39]. Autologous reconstruction, while involving larger wound surfaces and a higher risk of wound infection, generally results in complications that are easier to manage. Unlike alloplastic reconstruction, the most common complications of autologous reconstruction, such as delayed wound healing or infection, do not typically lead to severe consequences like failure of reconstruction [40].

Additionally, postoperative radiotherapy, often required for LABC patients, carries risks of poor wound healing. A recent systematic review comparing complications between immediate reconstruction and reconstruction after radiotherapy in patients undergoing autologous reconstruction found similar rates of flap loss, infection, fat necrosis, and wound healing complications [41], despite capsular contracture after IBR impact some aesthetic outcomes [42].

Autologous reconstruction is considered the gold standard for breast reconstruction, and is preferred in post-radiation patients [43, 44]. A retrospective cohort study highlighted the potential advantages of autologous reconstruction over alloplastic reconstruction, demonstrating a significantly lower reconstruction failure rate. However, no differences were observed in major complications or tumor outcomes between the two methods [45].While a prospective study showed that patients undergoing autologous reconstruction had better psychosocial well-being, despite the higher incidence of severe complications compared to alloplastic reconstruction [46]. Our further analysis of the effect of reconstruction methods on complications shows that autologous reconstruction had no advantage over alloplastic reconstruction in reducing surgical complications (OR: 0.80, 95%CI: [0.22,2.93], *p* = 0.73).

In terms of oncologic outcome, the present meta-analysis indicated that patients underwent IBR and patients underwent mastectomy alone were comparable in terms of overall survival and disease-free survival. Figure 2 shows that a few studies trended towards a worse OS outcome in IBR group, of which Di lenone A et al. reported a worse but non-statistically significant overall survival (HR:1.92, 95%CI: [0.69, 5.34], p = NS) [35], because of the relatively balanced T stage compared to other studies. The effect of IBR in LABC patients on survival has been investigated with controversial results in recent years. Sang et al. retrospectively compared 104 patients received IBR with 1715 patients underwent conventional mastectomy in a neoadjuvant chemotherapy cohort and found no statistical difference between groups related to DFS (HR: 0.72, *p* = 0.37) and LR (OR:2.83, *p* = 0.30) [37]. A single unit cohort study also demonstrated IBR may not compromise oncological and cosmetic outcomes with a low loco-regional recurrence (3.5%) or distant metastasis rate (3.2%) 1/28 after a median 61 months follow-up [17]. Conversely, several studies suggest that IBR should be carefully evaluated preoperatively. Di lenone A et al. reported a slightly worse overall survival (HR: 1.92, *p* = 0.21) comparing an oncoplastic surgery [34]. In terms of surgical process, there was no difference in positive margin rate (Fig. 4), suggesting that IBR, while aesthetic, did not affect the local-regional control of the surgery, which is consistent with local recurrence outcome.

With the limitations inherent in retrospective reviews and cosmetic procedures, enrolled patients were inevitably younger and had earlier stages [47], these imbalances in demographic factors and tumor characteristics may result in potential selection bias. However, survival outcomes were still comparable in the analysis comprising 3 matched cohort studies, while we assumed that the negative effects of IBR on survival need to be further assessed.

To conclude, although IBR in LABC patients does not prominently affect the oncology outcome of the patients, it brings an inevitably greater risk of complications and affects the follow-up treatment of the patients. Considering the long-term prognosis of the patients, we do not recommend IBR in LABC patients. Therefore, we suggest complication risks and ambiguous oncologic outcomes must be disclosed to patients during the initial consultation and surgeons should not encourage patients to sway IBR over conventional mastectomy arbitrarily in the future.

Our review has several limitations. Firstly, some demographic and tumor characteristics of patients were imbalanced, which may result in selection bias and selective reporting bias, these confounders constitute our evidence base and may limit interpretations. Secondly, there was little information about surgery available in primary articles, and the influence of surgery-related factors on outcome variables could not be further elaborated. We couldn’t determine whether it was a technical problem with the surgeon or the procedure itself that made the difference in complication risk. Thirdly, some HRs of survival measures were manually extracted from survival curves, resulting in inconsistent data sources. Fourthly, we failed to conduct the analysis of patient satisfaction because of the inconsistency and imprecision of different scales. In addition, the heterogeneity with respect to the type of mastectomy and reconstruction techniques may be a source of study bias, which is hard to generalize.

## Conclusion

Our updated meta-analysis is the first to evaluate oncologic outcomes and surgical complications in LABC patients underwent IBR compared to mastectomy. The review illustrates that patients underwent IBR has comparable overall survival, disease-free survival, and breast cancer specific survival to patients underwent mastectomy. Nevertheless, considering the significantly higher complication rates, IBR should be cautiously conducted. Further prospective, randomized studies with long-term follow-up are required to evaluate the survival outcomes of LABC patients underwent IBR.

### Electronic supplementary material

Below is the link to the electronic supplementary material.

Supplementary Material 1

Supplementary Material 2

## Data Availability

No datasets were generated or analysed during the current study.
