# Supplementary material for: Locally advanced breast cancer patients should be cautious about the immediate breast reconstruction after mastectomy: a pooling analysis of safety and efficacy
Source: World J Surg Oncol. 2024 Jun 25;22:165. doi: 10.1186/s12957-024-03444-z (PMC11197261; doi:10.1186/s12957-024-03444-z)
Supplement: Supplementary file 1 — Supplementary Material 1 [file 12957_2024_3444_MOESM1_ESM.docx]

Table S1. Characteristics of 3 studies under propensity score matching included in the meta-analysis.

| Characteristics | Studies | IBR/Non-IBR | Mean Difference/Odds Ratio (95% CI) | p | Heterogeneity |
| --- | --- | --- | --- | --- | --- |
| Histology grade | 3 |  | 0.99[0.89,1.11] | p=0.89 | I²=0%, P = 0.87 |
| I+II |  | 1273/2432 |  |  |  |
| III |  | 1597/2923 |  |  |  |
| Survival information |  |  |  |  |  |
| OS | 3 |  | 0.99[0.89,1.10] | p=0.87 | I²=39%, P = 0.19 |
| BCSS | 2 |  | 0.99[0.82,1.21] | p=0.95 | I²=62%, P = 0.11 |
