# Supplementary material for: Locally advanced breast cancer patients should be cautious about the immediate breast reconstruction after mastectomy: a pooling analysis of safety and efficacy
Source: World J Surg Oncol. 2024 Jun 25;22:165. doi: 10.1186/s12957-024-03444-z (PMC11197261; doi:10.1186/s12957-024-03444-z)
Supplement: Supplementary file 2 — Supplementary Material 2 [file 12957_2024_3444_MOESM2_ESM.docx]

1.
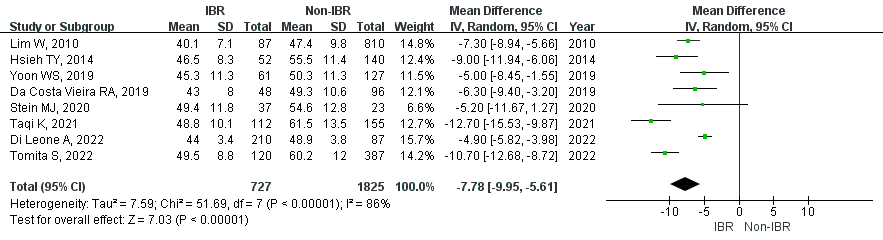

2.
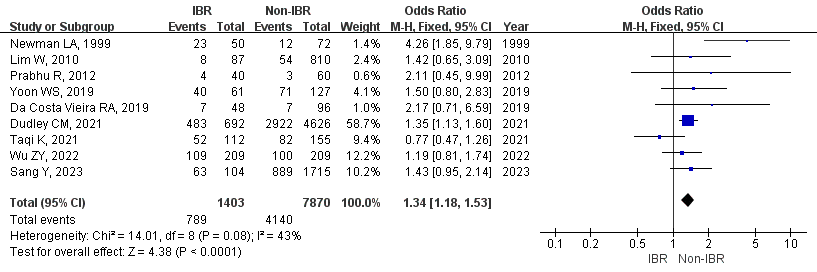

3.
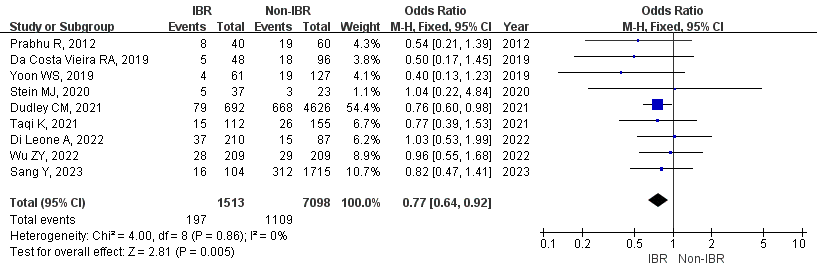

4.
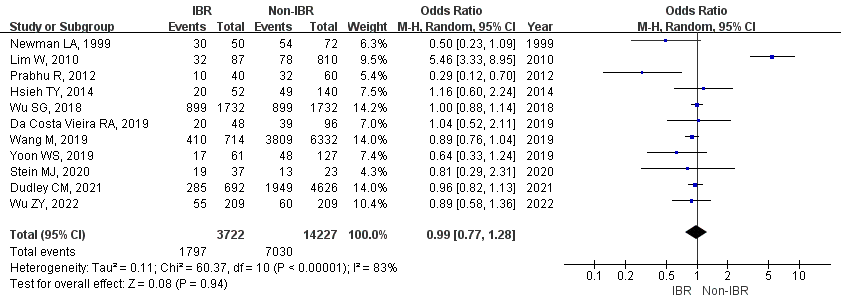


a. Age; b. Tumor stage; c. Molecular type; d. Histology grade.

Figure S1. Forest plot of tumor characteristics included in the study.
